# The effect of icodextrin on peritoneal dialysis patients with congestive heart failure: a time-varying exposure design and target trial emulation approach

**DOI:** 10.1093/ndt/gfaf265

**Published:** 2025-12-13

**Authors:** Li-Yi Ma, Yi-Ran Tu, Ming-Jen Chan, Chan Ip Chan, Jia-Jin Chen, Cheng-Chia Lee, Victor Chien-Chia Wu, Pao-Hsien Chu, Hsiang-Hao Hsu, Chih-Hsiang Chang

**Affiliations:** Department of Nephrology, Kidney Research Center, Chang Gung Memorial Hospital, Linkou Branch, Taoyuan, Taiwan; Department of Nephrology, Kidney Research Center, Chang Gung Memorial Hospital, Linkou Branch, Taoyuan, Taiwan; College of Medicine, Chang Gung University, Taoyuan, Taiwan; Department of Nephrology, Kidney Research Center, Chang Gung Memorial Hospital, Linkou Branch, Taoyuan, Taiwan; College of Medicine, Chang Gung University, Taoyuan, Taiwan; Medical Affairs, Vantive Healthcare Ltd, Taiwan; Department of Nephrology, Kidney Research Center, Chang Gung Memorial Hospital, Linkou Branch, Taoyuan, Taiwan; College of Medicine, Chang Gung University, Taoyuan, Taiwan; Department of Nephrology, Kidney Research Center, Chang Gung Memorial Hospital, Linkou Branch, Taoyuan, Taiwan; College of Medicine, Chang Gung University, Taoyuan, Taiwan; College of Medicine, Chang Gung University, Taoyuan, Taiwan; Department of Cardiology, Chang Gung Memorial Hospital, Linkou Branch, Taoyuan, Taiwan; College of Medicine, Chang Gung University, Taoyuan, Taiwan; Department of Cardiology, Chang Gung Memorial Hospital, Linkou Branch, Taoyuan, Taiwan; Institute of Stem Cell and Translational Cancer Research, Chang Gung Memorial Hospital, Taiwan; Department of Nephrology, Kidney Research Center, Chang Gung Memorial Hospital, Linkou Branch, Taoyuan, Taiwan; College of Medicine, Chang Gung University, Taoyuan, Taiwan; Department of Nephrology, Kidney Research Center, Chang Gung Memorial Hospital, Linkou Branch, Taoyuan, Taiwan; College of Medicine, Chang Gung University, Taoyuan, Taiwan

**Keywords:** heart failure, icodextrin, peritoneal dialysis

## Abstract

**Background:**

Icodextrin is an alternative peritoneal dialysis (PD) solution with favorable fluid management and metabolic properties. Given the limited evidence regarding the use of icodextrin in patients with pre-existing congestive heart failure (CHF), this study aimed to examine the association between icodextrin use and clinical outcomes in PD patients.

**Methods:**

We conducted a retrospective cohort study using the Chang Gung Research Database (CGRD), including 1800 eligible PD patients with CHF from 2005 to 2022 in Taiwan, followed through June 2023. Icodextrin users (≥50% of PD duration) were compared with non-users. Primary outcomes included all-cause mortality, cardiovascular mortality, sudden death and major adverse cardiovascular events (MACE). Multivariable Cox proportional hazards models incorporating time-varying exposure to icodextrin were applied. To evaluate the robustness of our primary analysis, we conducted a target trial emulation framework and an alternative time-dependent data structure as sensitivity analyses.

**Results:**

Icodextrin use was associated with lower risks of all-cause mortality [adjusted hazard ratio (HR) 0.16, 95% confidence interval (CI) 0.13–0.20], cardiovascular mortality (HR 0.20, 95% CI 0.13–0.30), sudden death (HR 0.15, 95% CI 0.11–0.19) and MACE (HR 0.68, 95% CI 0.58–0.80). Icodextrin users also showed associations with reduced risks of encapsulating peritoneal sclerosis and transition to hemodialysis but lower transplantation rates.

**Conclusions:**

In PD patients with CHF, icodextrin use was independently associated with better survival and cardiovascular outcomes. These findings support its preferential use in high-risk PD populations and warrant further prospective investigation.

## Graphical Abstract

**Graphical Abstract:**
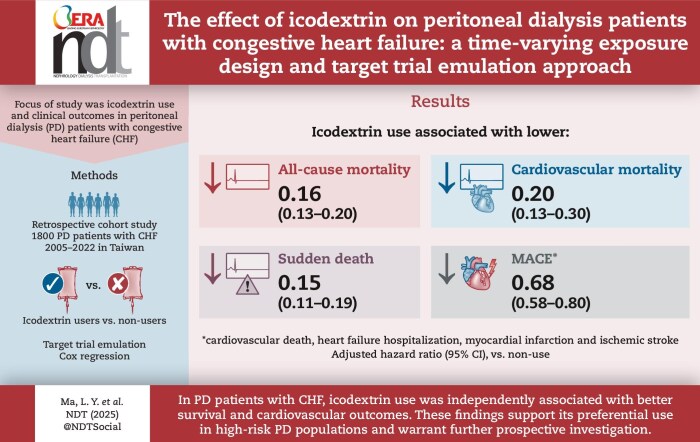

KEY LEARNING POINTS **What was known:**

- Congestive heart failure (CHF) is common in peritoneal dialysis (PD) patients and is associated with poor prognosis; effective fluid control is crucial for improving outcomes.
- Conventional glucose-based dialysate loses ultrafiltration efficiency during long dwells due to glucose absorption and may cause adverse metabolic effects.
- Icodextrin provides sustained ultrafiltration and reduces glucose load, offering potential benefits in volume-overloaded states, but evidence on hard cardiovascular outcomes in PD patients with pre-existing CHF is limited.

**This study adds:**

- In PD patients with pre-existing CHF, long-term icodextrin use was significantly associated with reduced risks of all-cause mortality, cardiovascular mortality and sudden death.
- Icodextrin use was linked to lower rates of major adverse cardiovascular events, with particularly pronounced benefits in patients with diabetes mellitus and those with moderate glycemic control (HbA1c 7%–9%).
- Icodextrin use was associated with reduced risks of encapsulating peritoneal sclerosis and transition to hemodialysis.

**Potential impact:**

- Findings support considering icodextrin as a preferred dialysate in high cardiovascular risk PD patients, especially younger individuals and those with diabetes mellitus, to improve long-term outcomes.
- Provides evidence to inform dialysate selection and volume management strategies, supporting more individualized treatment approaches.
- Future prospective studies could further validate clinical benefits and guide policy for broader adoption in appropriate patient populations.

## INTRODUCTION

Chronic kidney disease (CKD) affects 11%–13% of the global population and remains a major contributor to cardiovascular morbidity and premature death [1, 2]. Advances in healthcare and

increasing life expectancy have contributed to a growing population of patients progressing to end-stage kidney disease (ESKD), necessitating renal replacement therapy (RRT) [3]. Among available RRT modalities, peritoneal dialysis (PD) is widely utilized and offers several advantages over in-center hemodialysis (HD), including better preservation of residual renal function, more gradual and continuous solute and fluid removal, reduced cardiovascular stress, lower healthcare costs, greater treatment flexibility, improved quality of life and comparable survival outcomes [4–7]. Nevertheless, up to 40% of prevalent PD patients live with concomitant congestive heart failure (CHF), a combination that markedly worsens survival and quality of life [4]. Effective and sustainable volume control is therefore a cornerstone of PD management in this high-risk group.

Standard glucose-based dialysate achieves ultrafiltration by generating crystalloid osmotic gradients, yet its efficacy wanes during the long daytime dwell owing to systemic absorption of both water and glucose. Repeated exposure to high dialysate glucose concentrations is also implicated in peritoneal membrane deterioration, dysglycaemia and weight gain [5]. Icodextrin is a 7.5% iso-osmolar glucose polymer solution that exerts its dialytic effect through colloid rather than crystalloid osmosis, producing more constant ultrafiltration over 12- to 16-h dwells and enhancing sodium removal via convective drag [6]. Small randomized controlled trials and meta-analyses have reported favorable effects on left-ventricular mass index and blood pressure, but their sample sizes were modest and cardiovascular events were infrequent [8–10]. Consequently, major guidelines acknowledge the theoretical benefit of icodextrin in volume-expanded states but classify the evidence supporting hard outcomes as “low to moderate quality” [11–14] (Supplementary data, Table S1).

Previous studies have demonstrated that PD may serve as an effective therapeutic option for patients with CHF, improving cardiac functional status and reducing hospital admissions [15, 16]. Icodextrin has also been shown to lower the risk of new-onset CHF [17]. The present study aims to examine the associations between icodextrin use and all-cause mortality, cardiovascular mortality and major adverse cardiovascular events (MACE) in a multi-institutional cohort of PD patients with pre-existing CHF at the initiation of dialysis in institutions in Taiwan.

## MATERIALS AND METHODS

### Data source

This retrospective cohort study utilized data extracted from the Chang Gung Research Database (CGRD), a comprehensive electronic medical records repository derived from the Chang Gung Memorial Hospital (CGMH) system, the largest healthcare provider in Taiwan. The CGMH system comprises four tertiary referral centers and three academic hospitals, jointly accounting for nearly 10% of the nation’s medical services. The CGRD offers deidentified, patient-level data that includes information on prescriptions, outpatient and inpatient encounters, procedures, laboratory examinations and imaging studies, enabling high-quality longitudinal research [18, 19]. All potentially identifiable patients or provider information within the CGRD was anonymized through encryption and scrambling prior to data access, ensuring compliance with data privacy standards. Given the use of deidentified data, the requirement for written informed consent was waived. This study was approved by the Institutional Review Board of the Chang Gung Medical Foundation (IRB approval number: 202501239B0) and conducted in accordance with the ethical standards of the Declaration of Helsinki.

### Patient selection

**Figure 1:**
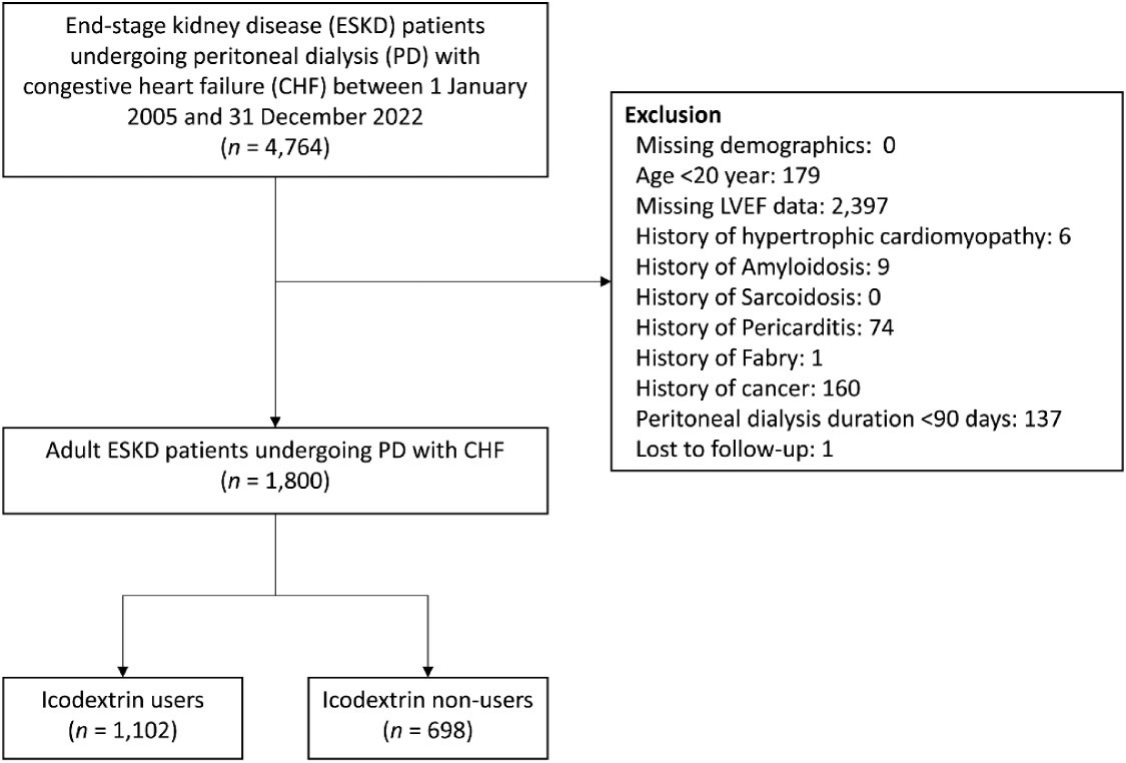
Patient inclusion–exclusion flowchart.

The study aimed to evaluate the clinical outcomes of icodextrin use in PD patients with pre-existing CHF. Patient selection is illustrated in Fig. 1. We identified adult patients (>20 years of age) with ESKD who initiated PD between 1 January 2005, and 31 December 2022. Eligible patients had to have undergone PD for at least 90 days and have a diagnosis of CHF. Patients with missing left ventricular ejection fraction (LVEF) data or a history of hypertrophic cardiomyopathy, amyloidosis, sarcoidosis, pericarditis, Fabry disease or malignancy were excluded.

### Exposure of icodextrin

Icodextrin users were defined as patients who received icodextrin for at least 50% of their total PD duration [7, 20]. According to Taiwan’s National Health Insurance regulations, icodextrin (EXTRANEAL, Vantive Health LLC) could be prescribed once daily for patients with glycated hemoglobin (HbA1c) >7.0%, those requiring 2.5% or 4.25% dextrose in more than half of their daily exchanges, or patients with high or high-average peritoneal membrane transporter status [7]. However, prescription decisions were ultimately at the discretion of treating physicians based on clinical needs for enhanced ultrafiltration or improved glycemic control.

Importantly, the use of icodextrin was treated as a time-varying exposure [7, 20]. For patients who never used icodextrin, their follow-up time consisted of a single segment. For those who initiated icodextrin at some point after starting PD and continued its use thereafter, their follow-up time was divided into two segments: an initial unexposed period followed by an exposed period. Furthermore, if a patient discontinued icodextrin after a period of use, the follow-up time was split into three segments: unexposed, exposed and unexposed again. If a patient initiated icodextrin use at the start of PD and continued without discontinuation, their follow-up time would consist of a single segment. If the patient later discontinued icodextrin, the follow-up time would be divided into two segments: the first segment being exposed and the second unexposed.

### Covariates

Covariates included demographic variables [age, sex, body mass index (BMI)], comorbidities, primary renal disease, dialysis modality, number of hospital admissions in the year prior to the index date, laboratory data, medication use and lifestyle factors (smoking, alcohol consumption), as well as the year of dialysis initiation and total follow-up duration.

Comorbidities included diabetes mellitus, hypertension, atrial fibrillation, peripheral artery disease, myocardial infarction, stroke, gout, liver cirrhosis, hepatitis B, hepatitis C and cardiovascular disease. Primary renal diseases included diabetic nephropathy, hypertensive nephropathy, chronic glomerulonephritis (including lupus nephritis, immunoglobulin A nephropathy and focal segmental glomerulosclerosis), adult polycystic kidney disease, obstructive nephropathy and interstitial nephritis. Comorbidities and primary renal disease were identified based on International Classification of Diseases (ICD), Clinical Modification codes (listed in Supplementary data, Table S2).

Laboratory values were derived from the most recent measurements within the 3 months preceding the index date and included serum albumin, hemoglobin, HbA1c, low-density lipoprotein, high-density lipoprotein, total cholesterol, triglycerides and uric acid. Concomitant medications were defined based on prescriptions within the 90 days preceding the index date and included high-potency statins, angiotensin-converting enzyme inhibitors (ACEi)/angiotensin receptor blockers (ARBs), beta-blockers, calcium channel blockers, loop diuretics, mineralocorticoid receptor antagonists (MRAs), nitrates, other vasodilators, angiotensin receptor-neprilysin inhibitors (ARNIs), antiplatelet agents, insulin and oral antidiabetic medications. It should be noted that most covariates were treated as time-varying, except for age, sex, modality and primary renal disease, which were considered fixed at baseline.

### Outcomes definition

The outcomes of primary interest were all-cause mortality, sudden death, MACE [defined as a composite of cardiovascular death, heart failure hospitalization (HFH), myocardial infarction and ischemic stroke] and cancer incidence. Secondary outcomes included renal complications: development of encapsulating peritoneal sclerosis, transition to hemodialysis and receipt of renal transplantation. Upon approval, death data were obtained from the Taiwan National Death Registry, managed by the Health and Welfare Data Science Center. Although the registry includes both primary and secondary causes of death, our access was limited to primary cause data. Patients who died within 90 days of switching from PD to hemodialysis were classified as having PD-related mortality [7]. HFH, myocardial infarction and ischemic stroke were captured based on hospitalization records. Censoring occurred at the time of encapsulating peritoneal sclerosis, transition to hemodialysis (if no death within 90 days), renal transplantation, the last visit of CGMHs or the end of the study period (30 June 2023), whichever came first.

### Statistical analysis

Baseline characteristics between icodextrin users and non-users were compared using chi-square tests for categorical variables and independent sample t-tests for continuous variables. The association between time-varying exposure to icodextrin and the risk of outcomes was assessed using Cox proportional hazards models. The models were adjusted for a set of covariates, including both time-invariant and time-varying factors. Time-invariant covariates included sex, age at PD initiation and the year of dialysis initiation. Time-varying covariates included BMI, diabetes mellitus, hypertension, cardiovascular disease, LVEF, dialysis modality, hospital admissions in the previous year, albumin, hemoglobin, use of high-potency statins, loop diuretics, insulin, cigarette smoking and alcohol consumption. Since missing values were present in variables such as BMI and laboratory results, single imputation was performed using the expectation-maximization (EM) algorithm.

Subgroup analyses were performed for the primary outcomes, including all-cause mortality, cardiovascular death, sudden death and MACE. These analyses were stratified by baseline characteristics: sex, age (<65 vs ≥65 years), BMI (<25 vs ≥25 kg/m²), presence of diabetes mellitus, cardiovascular disease, LVEF (≤60% vs >60%), dialysis modality, HbA1c level (<7%, 7%–9% and >9%), and duration from onset of CKD to PD initiation (<5 vs ≥5 years). Covariates were adjusted for when applicable in each subgroup analysis. In subgroup analyses, the main stratifying variable (e.g. BMI ≥25 kg/m²) was used in its original form without imputation, while covariates for adjustment were based on single EM-imputed data. All statistical tests were two-tailed, and *P* < .05 was considered statistically significant. Analyses were performed using SAS version 9.4 (SAS Institute, Cary, NC, USA).

### Sensitivity analyses

To evaluate the robustness of our primary analysis, we conducted two sensitivity analyses. First, we applied a target trial emulation framework. During the inclusion period (2005–22; a total of 18 years), a separate trial was emulated for each calendar quarter (1 January, 1 April, 1 July and 1 October), resulting in 72 emulated trials in total. In each quarterly trial, patients who were prescribed icodextrin were classified as the exposed group, while those who were not prescribed icodextrin during the same period served as the unexposed group, matched on age and sex frequency with 1:1 ratio. An intention-to-treat follow-up approach was adopted, with patients tracked until 30 June 2023, at the latest. Covariates included were identical to those used in the primary analysis, and robust standard errors were applied to adjust for the inflation of sample size resulting from repeated sampling across trials. This design minimizes the potential for immortal time bias to the greatest extent and provides the highest statistical power among the analytical approaches employed [21].

Second, we constructed an alternative time-dependent data structure beginning from each patient’s PD initiation date, dividing follow-up into 3-month observation intervals. Patients were classified as exposed during any interval in which icodextrin was prescribed, and as unexposed otherwise. For outcome-specific analyses, follow-up for each patient was terminated at the day of outcome occurrence. The covariates included were same as those in the primary analysis. This design may more accurately reflect real-world prescribing patterns, as patients often use icodextrin intermittently or for short periods in clinical practice [22].

## RESULTS

### Patient characteristics

A total of 1800 adult patients with ESKD undergoing PD and diagnosed with CHF were included in the study, comprising 1102 (61.2%) icodextrin users and 698 (38.8%) non-users (Fig. 1). Baseline demographic and clinical characteristics are summarized in Table 1 and Supplementary data, Table S3. Compared with non-users, icodextrin users were more likely to be male and had higher BMI values. Comorbid conditions such as diabetes mellitus and hypertension were also more prevalent among icodextrin users.

**Table 1:** Brief baseline demographics and clinical characteristics at PD initiation of patients with and without exposure to icodextrin.

Variable	Available number	Total (*n* = 1800)	Icodextrin users (*n* = 1102)	Icodextrin non-users (*n* = 698)	*P*-value
Male	1800	878 (48.8)	578 (52.5)	300 (43.0)	<.001
Age, years	1800	55.8 ± 15.1	55.4 ± 14.6	56.6 ± 15.9	.104
Body mass index, kg/m^2^	1669	24.3 ± 4.5	24.9 ± 4.6	23.2 ± 4.2	<.001
Comorbidity					
Diabetes mellitus	1800	859 (47.7)	637 (57.8)	222 (31.8)	<.001
Hypertension	1800	1627 (90.4)	1029 (93.4)	598 (85.7)	<.001
Atrial fibrillation	1800	93 (5.2)	64 (5.8)	29 (4.2)	.123
Peripheral artery disease	1800	126 (7.0)	74 (6.7)	52 (7.4)	.552
Myocardial infarction	1800	113 (6.3)	77 (7.0)	36 (5.2)	.119
Stroke	1800	146 (8.1)	93 (8.4)	53 (7.6)	.522
Cardiovascular disease	1800	604 (33.6)	386 (35.0)	218 (31.2)	.097
LVEF ≤60%	1800	584 (32.4)	380 (34.5)	204 (29.2)	.020
Primary renal disease	1800				<.001
Chronic tubulointerstitial disease		52 (2.9)	24 (2.2)	28 (4.0)	
Obstructive nephropathy		30 (1.7)	16 (1.5)	14 (2.0)	
Adult polycystic kidney disease		25 (1.4)	9 (0.8)	16 (2.3)	
Hypertension		912 (50.7)	523 (47.5)	389 (55.7)	
Diabetes mellitus		626 (34.8)	476 (43.2)	150 (21.5)	
Chronic glomerulonephritis		80 (4.4)	33 (3.0)	47 (6.7)	
Others		75 (4.2)	21 (1.9)	54 (7.7)	
Dialysis modality	1800				.037
APD		777 (43.2)	497 (45.1)	280 (40.1)	
CAPD		1023 (56.8)	605 (54.9)	418 (59.9)	
Admissions in the previous year	1800				.279
0		1588 (88.2)	965 (87.6)	623 (89.3)	
1–2		212 (11.8)	137 (12.4)	75 (10.7)	
Laboratory data at baseline					
Albumin, g/dL	1787	3.49 ± 0.53	3.46 ± 0.50	3.54 ± 0.57	.002
Hemoglobin, g/dL	1796	9.57 ± 1.42	9.49 ± 1.34	9.70 ± 1.52	.002
HbA1c, %	1386	6.1 ± 1.2	6.2 ± 1.2	5.8 ± 1.0	<.001
Cigarette smoking	1800	309 (17.2)	213 (19.3)	96 (13.8)	.002
Alcohol drinking	1800	158 (8.8)	105 (9.5)	53 (7.6)	.157
Duration from onset of CKD to PD, years	1476	4.2 ± 3.9	4.0 ± 3.8	4.5 ± 4.0	.024
Follow-up, years	1800	3.5 ± 3.1	3.6 ± 2.9	3.2 ± 3.2	.006

Data are presented as frequency (percentage), mean ± standard deviation or median (25th, 75th percentiles).

APD, automated peritoneal dialysis; CAPD, continuous ambulatory peritoneal dialysis.

Among icodextrin users, 310 patients (28.1%) initiated icodextrin use from the start of PD, whereas 792 patients (71.9%) began icodextrin treatment sometime after PD initiation. For the latter group, the median duration of PD prior to icodextrin initiation was 4.6 months (interquartile range 0.03–66.8 months), with a standard deviation of 7.6 months. Among icodextrin non-users (*n* = 698), 321 patients (46%) used icodextrin for <50% of their total PD duration (data not shown).

The majority of patients in both groups had heart failure with preserved ejection fraction. Approximately 67.6% of all patients had a LVEF >60%, while 32.4% had LVEF ≤60%, with no significant difference in LVEF distribution between the groups. Regarding underlying renal disease, diabetes mellitus was more frequently observed in icodextrin users, whereas hypertension, chronic glomerulonephritis and other etiologies were more common among non-users. Automated PD was more frequently utilized by icodextrin users.

The frequency of hospital admissions in the year prior to inclusion was comparable between groups. However, icodextrin users exhibited significantly lower baseline levels of serum albumin and hemoglobin, higher HbA1c and lower uric acid levels. Additionally, icodextrin users were more likely to be prescribed ACEi/ARBs, beta-blockers, calcium channel blockers, loop diuretics, MRAs, other vasodilators, antiplatelet agents, insulin and oral hypoglycemic agents. Cigarette smoking was more prevalent among icodextrin users. Initiation of PD occurred more frequently in the years 2011–22 among icodextrin users, while non-users more commonly initiated PD between 2005 and 2010. The mean follow-up duration was significantly longer for icodextrin users than for non-users (3.6 ± 2.9 vs 3.2 ± 3.2 years; *P* = .006).

### Outcomes

As shown in Table 2, icodextrin users had significantly lower incidences of all-cause mortality [3.2 vs 14.0 events per 100 person-years; adjusted hazard ratio (HR) 0.16, 95% confidence interval (CI) 0.13–0.20], cardiovascular mortality (1.0 vs 3.4 events per 100 person-years; adjusted HR 0.20, 95% CI 0.13–0.30), sudden death (2.3 vs 11.0 events per 100 person-years; adjusted HR 0.15, 95% CI 0.11–0.19) and MACE (9.9 vs 11.6 events per 100 person-years; adjusted HR 0.68, 95% CI 0.58–0.80) compared with non-users. There were no statistically significant differences between groups in the incidence of HFH (5.0 vs 3.7 events per 100 person-years; adjusted HR 1.06; *P* = .702), myocardial infarction (2.2 vs 1.6 events per 100 person-years; *P* = .785), ischemic stroke (1.8 vs 1.5 events per 100 person-years; *P* = .136) or new onset cancer (2.6 vs 2.8 events per 100 person-years; *P* = .458).

**Table 2:** Outcomes compared between icodextrin users and non-users in the primary analysis.

	Incidence^a^ (95% CI)		Crude analysis		Multivariable analysis^b^	
Outcome	Exposed	Unexposed	HR (95% CI)	*P*-value	HR (95% CI)	*P*-value
All-cause death	3.2 (2.6–3.8)	14.0 (12.6–15.4)	0.22 (0.18–0.27)	<.001	0.16 (0.13–0.20)	<.001
Cardiovascular death	1.0 (0.7–1.4)	3.4 (2.7–4.1)	0.28 (0.19–0.42)	<.001	0.20 (0.13–0.30)	<.001
Sudden death	2.3 (1.8–2.8)	11.0 (9.7–12.3)	0.20 (0.16–0.26)	<.001	0.15 (0.11–0.19)	<.001
Heart failure hospitalization	5.0 (4.2–5.7)	3.7 (3.0–4.5)	1.39 (1.07–1.80)	.015	1.06 (0.80–1.40)	.702
Myocardial infarction	2.2 (1.7–2.7)	1.6 (1.1–2.1)	1.36 (0.93–2.01)	.116	0.94 (0.63–1.43)	.785
Ischemic stroke	1.8 (1.4–2.2)	1.5 (1.0–2.0)	1.21 (0.81–1.82)	.356	1.39 (0.90–2.15)	.136
MACE^c^	9.9 (8.8–11.0)	11.6 (10.3–12.9)	0.83 (0.71–0.97)	.022	0.68 (0.58–0.80)	<.001
New diagnosis of cancer	2.6 (2.1–3.1)	2.8 (2.1–3.4)	0.87 (0.63–1.19)	.385	0.88 (0.63–1.23)	.458
Encapsulated peritoneal sclerosis	5.0 (4.3–5.7)	11.1 (10.1–12.2)	0.41 (0.35–0.49)	<.001	0.40 (0.33–0.48)	<.001
Shift to hemodialysis	13.0 (11.9–14.2)	22.1 (20.7–23.5)	0.59 (0.53–0.66)	<.001	0.52 (0.46–0.58)	<.001
Renal transplantation	1.5 (1.1–1.9)	5.1 (4.3–5.9)	0.25 (0.19–0.35)	<.001	0.22 (0.16–0.30)	<.001

^a^Number of events per 100 person-year.

^b^Adjusted for sex, age, body mass index, diabetes mellitus, hypertension, cardiovascular disease, LVEF, dialysis modality, admissions in the previous year, albumin, hemoglobin, high potency statin, loop diuretics, insulin, cigarette smoking, alcohol drinking and year of dialysis initiation.

^c^Any of cardiovascular death, heart failure hospitalization, myocardial infarction and ischemic stroke.

As detailed in Table 2, the risks of encapsulating peritoneal sclerosis (EPS) and transition to hemodialysis were lower in the icodextrin group compared with non-users (adjusted HR for EPS: 0.40, 95% CI 0.33–0.48; adjusted HR for transition to hemodialysis: 0.52, 95% CI 0.46–0.58). Conversely, icodextrin users showed a lower likelihood of undergoing renal transplantation (adjusted HR 0.22, 95% CI 0.16–0.30). An overview of outcome incidence is shown in Supplementary data, Table S4.

### Subgroup analyses

Subgroup analyses were performed to explore potential modifiers of the association between icodextrin use and major outcomes (Supplementary data, Fig. S1A–D). The associations between icodextrin use and lower risks of all-cause mortality, cardiovascular death, and sudden death were more pronounced in patients aged ≤65 years compared with those aged >65 years. Specifically, the HRs were 0.11 vs 0.25 for all-cause mortality, 0.10 vs 0.46 for cardiovascular death and 0.11 vs 0.20 for sudden death (all *P* for interaction <.05). The reduction in cardiovascular mortality was more evident in patients without pre-existing cardiovascular disease (HR 0.12 vs 0.33; *P* for interaction <.05), and those with baseline HbA1c levels between 7–9% (*P* for interaction <.05). In addition, the association with lower MACE risk was particularly notable among patients with diabetes mellitus (HR 0.57 vs 0.89; *P* for interaction <.05).

### Sensitivity analyses

In both sensitivity analyses, the beneficial association between icodextrin use and improved outcomes remained consistent with the primary findings. Under the target trial emulation framework (Table 3), icodextrin exposure was associated with markedly lower risks of all-cause mortality (adjusted HR 0.15, 95% CI 0.10–0.21), cardiovascular death (HR 0.11, 95% CI 0.05–0.26) and sudden death (HR 0.16, 95% CI 0.11–0.24) compared with non-users. Similarly, MACE were significantly reduced (HR 0.69, 95% CI 0.55–0.87), while no differences were observed for heart-failure readmission, myocardial infarction or ischemic stroke. Consistent patterns were observed in the alternative time-dependent model (Supplementary data, Table S5), in which icodextrin users demonstrated lower risks of all-cause mortality (HR 0.14, 95% CI 0.10–0.19), cardiovascular death (HR 0.08, 95% CI 0.04–0.18) and MACE (HR 0.69, 95% CI 0.57–0.84). Furthermore, the associations with reduced encapsulating peritoneal sclerosis and transition to hemodialysis remained significant across both analytic frameworks, reinforcing the robustness of the main results.

**Table 3:** Outcomes compared between icodextrin users and non-users with target trial emulation approach.

	Incidence^a^ (95% CI)		Crude analysis		Multivariable analysis^b^	
Outcome	Exposed (*n* = 11 879)	Unexposed (*n* = 11 879)	HR (95% CI)	*P*-value	HR (95% CI)	*P*-value
All-cause death	1.1 (0.7–1.5)	6.3 (5.4–7.3)	0.18 (0.12–0.26)	<.001	0.15 (0.10–0.21)	<.001
Cardiovascular death	0.2 (0.0–0.4)	1.5 (1.1–2.0)	0.14 (0.06–0.32)	<.001	0.11 (0.05–0.26)	<.001
Sudden death	1.0 (0.6–1.3)	5.0 (4.2–5.8)	0.19 (0.13–0.29)	<.001	0.16 (0.11–0.24)	<.001
Heart failure hospitalization	3.8 (3.1–4.6)	2.5 (1.9–3.2)	1.48 (1.08–2.04)	.015	1.11 (0.80–1.54)	.533
Myocardial infarction	1.6 (1.1–2.1)	1.2 (0.8–1.6)	1.40 (0.88–2.22)	.159	1.08 (0.67–1.74)	.754
Ischemic stroke	1.4 (1.0–1.9)	1.0 (0.6–1.4)	1.40 (0.86–2.29)	.173	1.21 (0.73–1.99)	.463
MACE^c^	6.0 (5.0–6.9)	6.4 (5.4–7.4)	0.92 (0.73–1.16)	.482	0.69 (0.55–0.87)	.002
New diagnosis of cancer	0.9 (0.6–1.3)	2.2 (1.6–2.7)	0.42 (0.27–0.67)	<.001	0.40 (0.25–0.63)	<.001
Encapsulated peritoneal sclerosis	2.4 (1.8–2.9)	3.9 (3.1–4.6)	0.62 (0.45–0.84)	.002	0.63 (0.45–0.86)	.004
Shift to hemodialysis	4.9 (4.1–5.8)	18.8 (16.6–21.0)	0.26 (0.22–0.32)	<.001	0.21 (0.17–0.26)	<.001
Renal transplantation	0.2 (0.0–0.4)	2.1 (1.5–2.6)	0.10 (0.04–0.24)	<.001	0.09 (0.04–0.21)	<.001

^a^Number of events per 100 person-year.

^b^Adjusted for sex, age, body mass index, diabetes mellitus, hypertension, cardiovascular disease, LVEF, dialysis modality, admissions in the previous year, albumin, hemoglobin, high potency statin, loop diuretics, insulin, cigarette smoking, alcohol drinking and year of dialysis initiation.

^c^Any of cardiovascular death, heart failure hospitalization, myocardial infarction and ischemic stroke.

## DISCUSSION

This retrospective cohort study evaluated the clinical outcomes associated with icodextrin use in PD patients with established CHF. Our findings demonstrate that icodextrin use is associated with lower risks of all-cause mortality, cardiovascular mortality, sudden death, MACE, EPS and transition to hemodialysis. Notably, these associations remained robust after adjustment for demographic, clinical, and laboratory covariates. The observed reduction in MACE risk appears to be primarily driven by a decrease in cardiovascular mortality, particularly sudden cardiac death.

The survival benefits associated with icodextrin use are consistent with its known pharmacological advantages, including enhanced ultrafiltration and reduced glucose exposure [5, 8, 14]. These properties may help support volume management and metabolic stability in PD patients with CHF, who frequently experience fluid overload and insulin resistance. The associations with lower cardiovascular and sudden death risks could plausibly reflect better hemodynamic stability and metabolic control [23].

**Figure 2:**
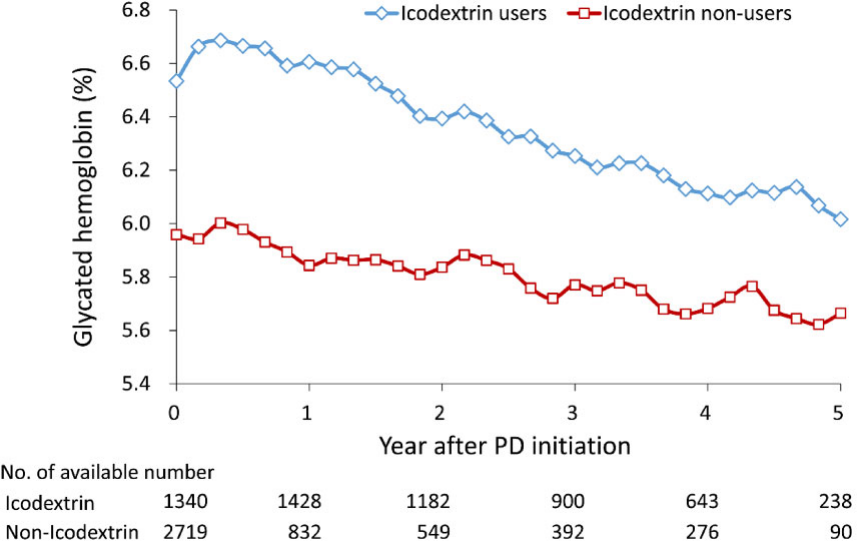
The changes in HbA1c during the period of icodextrin and glucose-based dialysate (non-icodextrin) exposure.

Icodextrin may also contribute to better glycemic control. Although icodextrin users had higher baseline HbA1c levels, HbA1c levels tended to decrease following PD initiation (Fig. 2). The optimal HbA1c target in diabetic patients undergoing dialysis remains controversial. While a general target of <7% is often recommended for adults with diabetes, some studies suggest that an HbA1c range of 6%–8% or 7%–9% may be associated with better survival in this population [24–28]. Our subgroup analyses support this notion: icodextrin users with moderate glycemic control (HbA1c 7%–9%) showed the strongest associations with lower cardiovascular risk. Although no significant differences in all-cause mortality were found in these subgroups, a trend toward improved outcomes in patients with moderate glycemic control was observed. Furthermore, patients with diabetes mellitus exhibited stronger associations with lower MACE risk, suggesting potential cardiometabolic advantages of icodextrin in high-risk populations.

Subgroup analyses also indicated that younger patients (≤65 years) and those without preexisting cardiovascular disease demonstrated stronger associations with lower cardiovascular risks in relation to icodextrin use. These findings suggest that early intervention with icodextrin, particularly in younger patients or those with less advanced cardiovascular disease, may be linked to more favorable clinical outcomes.

Our study also observed an association between icodextrin use and a lower risk of EPS. However, some retrospective studies have reported a potential positive association between icodextrin-based solutions and increased EPS risk [29]. While icodextrin has a lower glucose degradation product concentration, suggesting better biocompatibility and potentially lower EPS risk [8, 30, 31], other data suggest a complex relationship. For instance, Lopez-Anton *et al*. [32] reported that icodextrin use may promote miR-21 expression in mesothelial cells, contributing to peritoneal fibrosis and possibly EPS. It is noteworthy that icodextrin is often prescribed in patients with ultrafiltration failure and prolonged PD vintage, both of which are independent risk factors for EPS [29, 33]. Morelle *et al*. [34] further reported that early ultrafiltration failure is a hallmark of EPS development, independent of icodextrin use. Therefore, the association between icodextrin and EPS warrants further investigation.

Interestingly, although icodextrin use was associated with lower rates of EPS and transition to hemodialysis, it was also linked to a reduced likelihood of kidney transplantation. This finding may reflect unmeasured clinical or socioeconomic factors affecting transplant eligibility, such as the scarcity of living donors in Taiwan or clinician preferences. Further studies are needed to explore these potential confounders.

No significant differences were observed between icodextrin users and non-users in the risks of cancer, myocardial infarction, ischemic stroke or HFH. These neutral findings suggest that while icodextrin use was associated with lower overall mortality and cardiovascular burden, it did not appear to have a measurable relationship with atherosclerotic events or malignancies.

The consistency of findings across sensitivity analyses further strengthens the robustness of our observations. Both the target trial emulation and the time-dependent exposure models yielded HRs closely aligned with those of the primary Cox analysis, suggesting that the survival and cardiovascular benefits of icodextrin are unlikely to be explained by immortal-time or exposure-misclassification bias. The target trial emulation design, which mimics randomization by matching patients on age and sex within quarterly enrollment cohorts, minimizes temporal confounding and supports a more reliable interpretation of the association between icodextrin use and reduced mortality. Similarly, the time-dependent exposure approach, incorporating 3-month observation intervals, captures real-world prescribing patterns and intermittent use common in PD practice. The persistence of benefit across these complementary methods indicates that icodextrin’s favorable effect on survival and cardiovascular outcomes is both statistically and clinically robust, underscoring its potential as a preferred dialysate for patients with coexisting congestive heart failure.

This study has several strengths, including the use of a comprehensive clinical database with granular laboratory data and echocardiographic parameters, and the application of a time-dependent Cox model to account for treatment exposure changes over time. However, several limitations should be noted. First, using ICD codes for patient diagnosis and screening may miss some cases for conditions not coded correctly. Second, as an observational study, causality cannot be established, and residual confounding may persist despite multivariate adjustment. Third, the choice to prescribe icodextrin was not randomized and may reflect clinician judgment based on clinical severity or patient characteristics not captured in the dataset. In addition, the reimbursement criteria of Taiwan’s National Health Insurance imposed limitations on the study. Finally, although we included a broad spectrum of covariates, some potentially relevant variables such as socioeconomic status, dietary adherence and physical activity were unavailable.

Future research may consider conducting multicenter randomized controlled trials that incorporate volume status assessment tools, such as bioimpedance spectroscopy, and biomarkers like B-type natriuretic peptide, to better evaluate their roles in guiding fluid management and improving cardiovascular and renal outcomes. In addition, the application of cost-effectiveness modeling may help assess the economic and clinical value of various monitoring and treatment strategies in patients undergoing PD. Further subgroup analyses focusing on individuals with high glycemic burden or underlying diabetes could also provide important insights into differential therapeutic responses and optimize individualized treatment approaches.

## CONCLUSION

Icodextrin use in PD patients with CHF is associated with more favorable cardiovascular and renal outcomes, particularly among younger individuals and those with diabetes mellitus. These findings support the consideration of icodextrin as a preferred dialysate in this high-risk population and underscore the need for prospective trials to further validate its long-term clinical advantages.

## Supplementary Material

gfaf265_Supplemental_File

## Data Availability

The data underlying this article will be shared on reasonable request to the corresponding author.
