# Supplementary material for: The effect of icodextrin on peritoneal dialysis patients with congestive heart failure: a time-varying exposure design and target trial emulation approach
Source: Nephrol Dial Transplant. 2025 Dec 13;41(7):1313–21. doi: 10.1093/ndt/gfaf265 (PMC13314375; doi:10.1093/ndt/gfaf265)
Supplement: gfaf265_Supplemental_File [file gfaf265_supplemental_file.pdf]

Supplementary Figure S1.

Subgroup analysis of **(A)** all-cause mortality, **(B)** cardiovascular death, **(C)** sudden death and **(D)** major adverse cardiovascular events stratified by baseline characteristics. Subgroup analyses included covariate adjustment where applicable. Stratifying variables were used in their original form without imputation, whereas adjustment covariates were derived from imputed data.

(A)

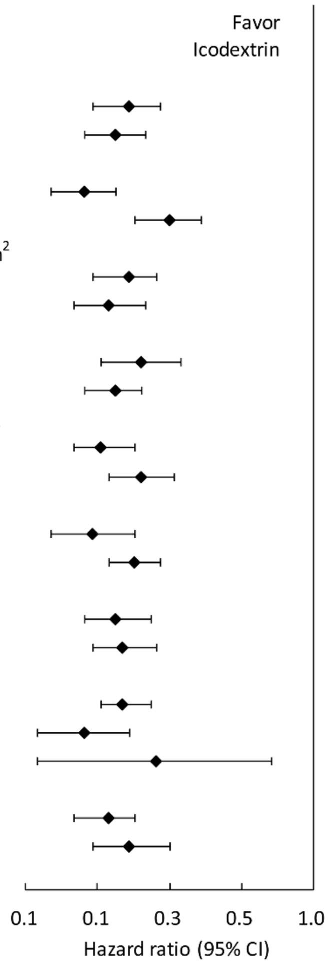

Subgroup	Incidence Icodextrin	Incidence Non-Icodextrin	HR (95% CI) for Icodextrin	*P* value for interaction
Sex				0.655
Female	2.8	5.3	0.17 (0.12–0.23)	
Male	3.1	6.0	0.15 (0.11–0.20)	
Age, year				<0.001
≤65	1.7	3.7	0.11 (0.08–0.15)	
>65	9.5	14.5	0.25 (0.18–0.34)	
Body mass index, Kg/m^2				0.49
<25	2.5	5.5	0.17 (0.12–0.22)	
≥25	3.9	6.1	0.14 (0.10–0.20)	
Diabetes mellitus				0.308
No	1.4	3.4	0.19 (0.13–0.28)	
Yes	4.8	10.6	0.15 (0.11–0.19)	
Cardiovascular disease				0.104
No	1.9	4.2	0.13 (0.10–0.18)	
Yes	5.8	9.7	0.19 (0.14–0.26)	
LVEF, %				0.098
≤60	2.8	5.7	0.12 (0.08–0.18)	
>60	3.1	5.6	0.18 (0.14–0.23)	
Dialysis modality				0.77
APD	3.0	6.0	0.15 (0.11–0.21)	
CAPD	3.0	5.4	0.16 (0.12–0.22)	
HbA1c, %				0.253
<7	2.3	4.9	0.16 (0.13–0.21)	
7-9	7.0	14.9	0.11 (0.07–0.17)	
>9	6.6	12.2	0.22 (0.07–0.67)	
CKD follow-up year				0.333
<5	2.9	5.6	0.14 (0.10–0.18)	
≥5	4.3	7.4	0.17 (0.12–0.25)	

(B)

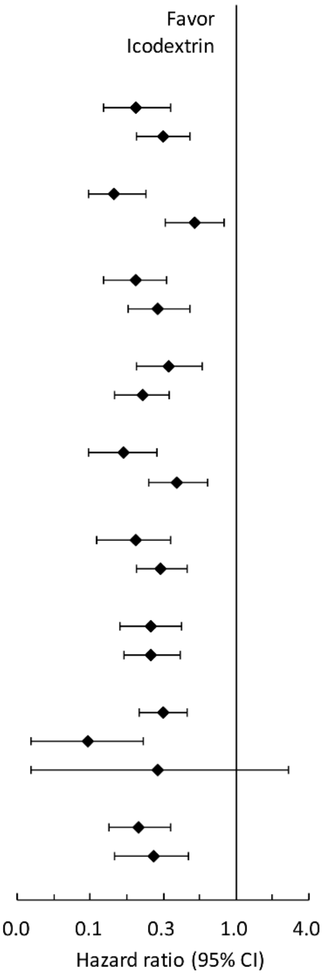

Subgroup	Incidence: Icodextrin	Incidence: Non-Icodextrin	HR (95% CI) for Icodextrin	*P* value for interaction
Sex				0.229
Female	0.7	1.3	0.15 (0.08–0.29)	
Male	1.2	1.4	0.25 (0.15–0.42)	
Age, year				<0.001
≤65	0.5	1.1	0.10 (0.06–0.18)	
>65	3.5	2.6	0.46 (0.26–0.81)	
Body mass index, Kg/m^2				0.301
<25	0.6	1.4	0.15 (0.08–0.27)	
≥25	1.5	1.4	0.23 (0.13–0.42)	
Diabetes mellitus				0.212
No	0.6	0.9	0.28 (0.15–0.53)	
Yes	1.4	2.4	0.17 (0.10–0.28)	
Cardiovascular disease				0.013
No	0.5	1.1	0.12 (0.06–0.22)	
Yes	2.2	2.1	0.33 (0.19–0.58)	
LVEF, %				0.223
≤60	1.0	1.5	0.15 (0.07–0.29)	
>60	1.0	1.3	0.24 (0.15–0.40)	
Dialysis modality				0.945
APD	1.1	1.5	0.20 (0.11–0.36)	
CAPD	0.9	1.3	0.20 (0.12–0.35)	
HbA1c, %				0.04
<7	0.9	1.2	0.25 (0.16–0.39)	
7-9	1.1	3.6	0.06 (0.02–0.17)	
>9	1.3	2.2	0.23 (0.02–2.73)	
CKD follow-up year				0.599
<5	0.8	1.3	0.16 (0.09–0.29)	
≥5	1.5	2.0	0.21 (0.10–0.41)	

(C)

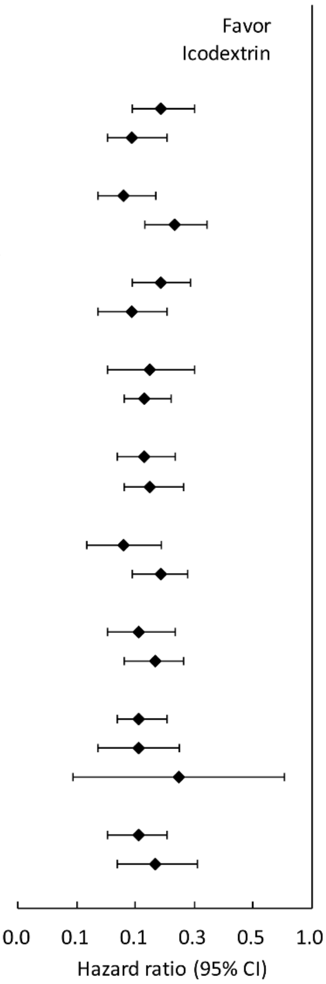

Subgroup	Incidence Icodextrin	Incidence Non-Icodextrin	HR (95% CI) for Icodextrin	*P* value for interaction
Sex				0.172
Female	2.2	4.1	0.17 (0.12–0.25)	
Male	2.0	4.8	0.12 (0.09–0.18)	
Age, year				0.014
≤65	1.3	2.8	0.11 (0.08–0.16)	
>65	6.5	12.1	0.20 (0.14–0.29)	
Body mass index, Kg/m^2				0.146
<25	2.0	4.4	0.17 (0.12–0.24)	
≥25	2.5	4.9	0.12 (0.08–0.18)	
Diabetes mellitus				0.87
No	0.8	2.6	0.15 (0.09–0.25)	
Yes	3.7	8.5	0.14 (0.11–0.19)	
Cardiovascular disease				0.697
No	1.5	3.2	0.14 (0.10–0.20)	
Yes	3.8	7.9	0.15 (0.11–0.22)	
LVEF, %				0.119
≤60	1.9	4.4	0.11 (0.07–0.17)	
>60	2.3	4.4	0.17 (0.12–0.23)	
Dialysis modality				0.52
APD	2.0	4.6	0.13 (0.09–0.20)	
CAPD	2.2	4.3	0.16 (0.11–0.22)	
HbA1c, %				0.765
<7	1.5	3.9	0.13 (0.10–0.18)	
7-9	6.2	11.3	0.13 (0.08–0.21)	
>9	5.3	10.0	0.21 (0.06–0.73)	
CKD follow-up year				0.402
<5	2.2	4.5	0.13 (0.09–0.18)	
≥5	3.0	5.5	0.16 (0.10–0.26)	

(D)

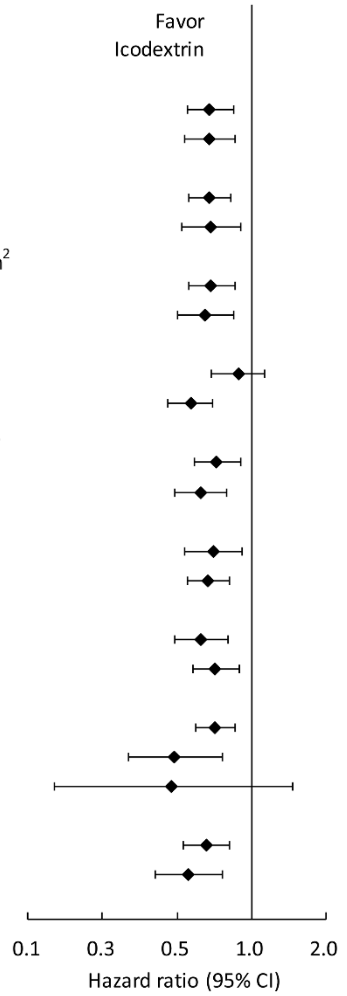

Subgroup	Incidence Icodextrin	Incidence Non-Icodextrin	HR (95% CI) for Icodextrin	*P* value for interaction
Sex				0.998
Female	8.6	5.7	0.68 (0.55–0.85)	
Male	9.7	5.8	0.68 (0.54–0.86)	
Age, year				0.945
≤65	7.8	4.5	0.68 (0.56–0.83)	
>65	15.8	11.3	0.69 (0.52–0.91)	
Body mass index, Kg/m^2				0.725
<25	8.5	5.6	0.69 (0.56–0.86)	
≥25	10.5	6.4	0.65 (0.50–0.85)	
Diabetes mellitus				0.006
No	6.3	3.8	0.89 (0.69–1.13)	
Yes	12.3	10.1	0.57 (0.46–0.70)	
Cardiovascular disease				0.348
No	6.9	4.0	0.73 (0.59–0.91)	
Yes	15.6	11.0	0.63 (0.49–0.80)	
LVEF, %				0.752
≤60	12.9	7.0	0.71 (0.54–0.92)	
>60	7.5	5.2	0.67 (0.55–0.82)	
Dialysis modality				0.432
APD	9.9	6.4	0.63 (0.49–0.81)	
CAPD	8.6	5.4	0.72 (0.58–0.89)	
HbA1c, %				0.227
<7	8.5	5.3	0.72 (0.60–0.86)	
7-9	13.7	11.3	0.49 (0.32–0.76)	
>9	7.6	8.0	0.48 (0.16–1.48)	
CKD follow-up year				0.381
<5	9.6	5.8	0.66 (0.53–0.82)	
≥5	8.9	7.3	0.56 (0.41–0.76)	

HR, hazard ratio; CI, confidence interval; CVD, cardiovascular disease; LVEF, left ventricular ejection fraction; APD, automated peritoneal dialysis; CAPD, continuous ambulatory peritoneal dialysis; HbA1c, glycated hemoglobin; CKD, chronic kidney disease.

Supplementary Table S1. Literature review of icodextrin versus glucose solutions

Year	Author	Objective	Study Design	Case Numbers	Conclusion(s)
1994	Mistry et al.[1][†,‡,#]	To evaluate the long-term safety and efficacy by comparing daily overnight (8 to 12 hr dwell) use of isosmolar icodextrin with conventional 1.36% and 3.86% glucose exchanges over six months	RCT, multicenter study	ICO, n=106<br>GLU, n=103	The daily overnight use of an isosmolar icodextrin solution was safe and effective up to six months and could replace the overnight use of hyperosmolar glucose solutions.
1995	Gokal et al.[2]	To compare peritonitis occurrence and outcome in a large U.K. study [Multicentre Investigation of Icodextrin in Ambulatory Dialysis (MIDAS)]	RCT	ICO, n=106<br>GLU, n=103	Using icodextrin for the long dwell in CAPD does not increase the rate of peritonitis, nor does it alter the outcome of peritonitis. Peritonitis does not affect uptake of icodextrin from the peritoneum.
1997	Posthuma et al.[3][‡,#]	Icodextrin instead of glucose solutions during the daytime dwell in CCPD patient	RCT	ICO, n=12<br>GLU, n=11	Icodextrin enhances ultrafiltration during the daytime dwell in CCPD patients.
1997	Posthuma et al.[4][‡,#]	To evaluate the safety, efficacy, and biocompatibility of icodextrin- and glucose-	RCT, two-center study	ICO, n=12<br>GLU, n=13	The serum icodextrin metabolite levels increased markedly and were the same as those found previously in CAPD patients treated with icodextrin, despite the longer dwell time for CCPD patients (14-16 hr versus 8-

Year	Author	Objective	Study Design	Case Numbers	Conclusion(s)
		containing dialysis fluid during CCPD			12 hr). The initial decrease in serum sodium concentration was followed by an increase to a concentration not different from baseline at 12 months.<br>Calculated osmolal gap in icodextrin patients increased significantly at every follow-up visit, and could be explained by the serum icodextrin metabolite increase.<br>No clinical side effects were observed despite increased levels of icodextrin metabolites.
2000	Posthuma et al.[5][†,#]	Assess the efficacy, safety, and biocompatibility of icodextrin solution compared to glucose solution as the daytime dwell in continuous cycling peritoneal dialysis	RCT	ICO, n=19<br>GLU, n=19	The use of icodextrin for the long daytime dwell in CCPD led to an increase in total UF of at least 261 mL per day, which was maintained over at least 24 months.<br>During icodextrin treatment, serum icodextrin metabolites increased significantly and serum sodium concentrations decreased initially. As a result, serum osmolality increased slightly.
2001	Bredie et al.[6][‡,#]	Effects of an overnight icodextrin dwell on parameters of glucose and lipid metabolism	Randomized crossover study	21 non-diabetic CAPD patients	There is a beneficial effect on lipid profiles of CAPD patients with the use of an overnight dwell with icodextrin.

Year	Author	Objective	Study Design	Case Numbers	Conclusion(s)
2002	Plum et al.[7][†,‡,#]	7.5% icodextrin solution compared to 2.27% glucose solution in patients undergoing APD	RCT, multicenter study	ICO, n=17<br>GLU, n=16	Icodextrin produced increased, sustained ultrafiltration during the long dwell period, increasing peritoneal clearance and sodium removal in automated peritoneal dialysis patients.<br>Urine volume and residual renal function were not specifically affected by icodextrin compared with glucose.
2002	Wolfson et al.[8][†,‡,#]	Compare the efficacy and long-term safety of icodextrin and 2.5% dextrose for the once-daily long dwell in CAPD and APD	2 Double-blind RCTs	Efficacy study:<br>ICO, n=81<br>GLU, n=82;<br>Safety study:<br>ICO, n=106<br>GLU, n=63	Icodextrin provides patients with greater fluid removal and small solute clearance, no weight gain over 52 weeks, and a decreased risk of edema.
2003	Davies et al.[9][†,‡,#]	Effects of Icodextrin compared to 2.27% glucose solution	Double-blind RCT	ICO, n=28<br>GLU, n=22	The use of icodextrin in the long exchange improves fluid removal and status in PD, without harmful effects on residual renal function.
2003	Konings et al.[10][†,‡,#]	Effects of icodextrin 7.5% solution on fluid status, blood pressure regulation, and	RCT	ICO, n=22<br>GLU, n=18	The use of icodextrin resulted in a significant reduction in ECW and LVM.<br>The effect of icodextrin on ECW was not related to

Year	Author	Objective	Study Design	Case Numbers	Conclusion(s)
		echocardiographic parameters			peritoneal membrane characteristics, but was related to the initial fluid state of the patient.
2005	Finkelstein et al. [11][†,‡,#]	Comparing icodextrin and 4.25% dextrose during the long dwell of automated peritoneal dialysis	Double-blind RCT, multicenter study	ICO, n=47<br>GLU, n=45	In APD patients with high-average or high transport characteristics, icodextrin for the long-dwell exchange results in significantly greater net UF than 4.25% dextrose.<br>Significant improvements in the amount of fluid removed per gram of carbohydrate absorbed from dialysate indicated that icodextrin is a more efficient osmotic agent.
2007	Rodríguez-Carmona et al.[12][#]	To analyze the effects of icodextrin-based dialysate in the nocturnal schedule of patients undergoing APD	Randomized crossover study	17 APD patients	Inclusion of amino acid- and icodextrin-based solutions in the nocturnal schedule of APD patients may allow sustained ultrafiltration and sodium removal while significantly reducing the peritoneal glucose load in these patients.
2008	Davies et al.[13][‡]	Effects of Icodextrin compared to 2.27% glucose solution on fluid status, inflammation, urine volume and plasma metabolites	RCT, longitudinal observation	ICO, n=28<br>GLU, n=22	Changes in fluid status are associated with changes in urine volume; Icodextrin was not associated with a greater fall in urine output despite its larger effect on ECFv.<br>Changes in fluid status did not appear to influence systemic inflammation.

Year	Author	Objective	Study Design	Case Numbers	Conclusion(s)
2008	Paniagua et al.[14][‡]	Effects of icodextrin solutions on echocardiographic, electrocardiographic, and blood pressure changes in diabetic patients on PD	RCT	ICO, n=30<br>GLU, n=29	Icodextrin solution exerted mixed effects in diabetic PD patients; some of them, such as lowering BP and NT-pro-BNP or TNT values, seem to be volume-related. Changes in sympathetic tone expressed by LF R-R variability were likely derived from a better metabolic control.
2009	Lin et al.[15][†,‡,#]	Effects of nocturnal icodextrin compared to 2.5% glucose solution	Double-blind RCT	ICO, n=98<br>GLU, n=103	Icodextrin significantly improved ultrafiltration and small solute clearance, even in patients with low-average peritoneal transport.
2009	Paniagua et al.[16][†,#]	To analyze the effects of ICO on metabolic and fluid control in high and high-average transport diabetic patients on CAPD	RCT, multicenter study	ICO, n=30<br>GLU, n=29	Icodextrin represents a significant advantage in the management of high transport diabetic patients on PD, improving peritoneal UF and fluid control and reducing the burden of glucose overexposure, thereby facilitating metabolic control.
2011	Qi et al.[17]	To compare icodextrin and glucose for the once-daily long dwell in PD	Meta-analysis of RCTs	9 RCTs, n=1190	Icodextrin provides patients with greater fluid removal and small solute clearance and does not cause any damage to residual renal function.<br>Icodextrin is particularly appropriate for use in patients with high peritoneal transport status.
2011	Takatori et al.[18][‡,#]	To measure the beneficial effects of icodextrin on	RCT	ICO, n=21<br>GLU, n=20	In PD therapy for diabetic nephropathy, the use of icodextrin-containing solutions has a beneficial effect

Year	Author	Objective	Study Design	Case Numbers	Conclusion(s)
		technique failure, body fluid management, glucose and lipid metabolism, and residual renal and peritoneal functions of PD therapy			on technique survival, but there are no apparent benefits or disadvantages in residual renal and peritoneal functions compared with conventional PD with glucose solution.
2012	Han et al.[19]	Icodextrin group compared to non-icodextrin group on all-cause mortality and technique failure rates	Retrospective cohort from database	ICO, n=641 GLU, n=1522	Icodextrin solution improved patient's survival and technique success.
2014	Chow et al.[20][#]	Effects of icodextrin on the treatment of peritoneal dialysis patients during acute peritonitis	RCT	ICO, n=22 GLU, n=31	Use of icodextrin achieved better ultrafiltration and fluid control during acute peritonitis complicating continuous ambulatory peritoneal dialysis. No evidence of a worthwhile clinical benefit on peritonitis resolution.
2014	Sniderman et al.[21]	To determine whether a low-glucose PD regimen may improve the serum lipid and lipoprotein profile in patients with diabetes	RCT, multinational study	ICO, n=124 GLU, n=127	A low glucose-PD regimen significantly improved the atherogenic lipoprotein phenotype compared with PD patients treated with a conventional glucose regimen.
2014	Yoon et al.[22][#]	CAPD patients using one icodextrin-containing and	RCT	ICO, n=41 GLU, n=39	The CAPD technique using one icodextrin-containing and two glucose-containing dialysates tends to better

Year	Author	Objective	Study Design	Case Numbers	Conclusion(s)
		two glucose-containing dialysates a day versus all glucose-containing dialysates			preserve RRF and is more biocompatible, with similar dialysis adequacy compared to that using four glucose-containing dialysates in incident CAPD patients.
2015	de Moraes et al.[23][#]	To analyze effect of the substitution of glucose for icodextrin on insulin resistance in non-diabetic PD patients	RCT, multicenter study	ICO, n=33<br>GLU, n=27	The substitution of glucose for icodextrin for the long dwell improved insulin resistance measured by HOMA index in non-diabetic APD patients.
2016	Chang et al.[24][#]	Icodextrin solution versus glucose solution on the preservation of RRF in PD patients	RCT, multicenter study	ICO, n=49<br>GLU, n=51	Icodextrin solution preserves residual urine volume better than glucose solution.
2018	Chen et al.[25]	Compare the impact of ICO- and GLU-based solutions on cardiac structure and function in a 2-year longitudinal period in incident-APD patients	RCT	ICO, n=20<br>GLU, n=18	Long-dwell icodextrin solution can maintain reasonable cardiac structure and function in incident-APD patients.
2018	Htay et al.[26]	Biocompatible PD solutions in comparison to standard PD	Cochrane database systemic review	13 studies[*], n=1291	Icodextrin probably reduced episodes of uncontrolled fluid overload and augmented peritoneal ultrafiltration without compromising RRF which approximated to a

Year	Author	Objective	Study Design	Case Numbers	Conclusion(s)
		solutions in patients receiving PD			mean creatinine clearance of 0.30 mL/min/1.73m^2 higher or urine output.<br>It is uncertain whether icodextrin use led to any differences in adverse events technique failure or death.
2018	Wang et al.[27]	Use icodextrin ≥30 days compared to never use icodextrin on new onset CHF	Retrospective cohort from database	ICO, n=2931<br>GLU, n=2531	Icodextrin solution could reduce the risk of new-onset CHF, particularly effective when diabetic PD patients use it.
2020	Goossen et al.[28]	Once-daily long-dwell icodextrin compared to glucose solutions	Systemic review of RCTs	19 RCTs, n=1693	High certainty: Icodextrin-containing PD improved ultrafiltration and fewer episodes of fluid overload occurred; Icodextrin-containing PD did not directly change in fasting glucose and HbA1c levels.<br>Moderate certainty: Icodextrin-containing PD probably decreased mortality risk.<br>Safety outcomes and residual kidney function were similar in both groups.
2022	Cordeiro et al.[29]	Effects of Icodextrin compared to glucose solutions on LVM	RCT	ICO, n=10<br>GLU, n=12	Icodextrin-based solution compared with glucose-based solution was not able to improve LVM.
2024	Azevedo et al.[30]	Icodextrin versus glucose 2.5% on markers of	RCT	ICO, n=15<br>GLU, n=15	Icodextrin significantly improved ultrafiltration, extracellular water, and phase angle at the end of the

Year	Author	Objective	Study Design	Case Numbers	Conclusion(s)
		hypervolemia and survival of patients undergoing APD with an unplanned start			study compared to baseline in patients on the urgent start of automated peritoneal dialysis.
2024	Wang et al.[31]	Effects of Icodextrin compared to glucose solutions on the risks of death, technique failure and the first episode of peritonitis	Retrospective cohort from database; Time-varying study	ICO, n=190 GLU, n=535	Icodextrin users had significant lower risks of mortality, technique failure, and the first peritonitis episode. The risk of peritonitis reduced further in icodextrin users with diabetes and with cardiovascular disease.

Abbreviations: RCT, randomized controlled trial; hr, hour(s); ICO, icodextrin group; GLU, glucose solution group; CAPD, continuous ambulatory peritoneal dialysis; CCPD, continuous cycling peritoneal dialysis; UF, ultrafiltration; APD, automated peritoneal dialysis; PD, peritoneal dialysis; ECW, extracellular water; LVM, left ventricular mass; ECFv, volume of extra-cellular fluid; BP, blood pressure; NT-pro-BNP, N-terminal pro-brain natriuretic peptide; TNT, troponin T; LF, power in the low frequency domain; RRF, residual renal function; HOMA, homeostatic model assessment; CHF, congestive heart failure.

† Studies included in reference 17.

‡ Studies included in reference 26.

# Studies included in reference 28.

* Only studies related to glucose polymer (icodextrin) versus standard glucose dialysate are included. Studies related to neutral pH, low GDP solution versus standard glucose dialysate are not included.

Supplementary Table S2. Disease code used in this study

Disease	ICD-9-CM disease code	ICD-10-CM disease code
Heart failure	428.x	I50
Diabetes mellitus	250.x	E08–E13
Hypertension	401.x–405.x	I10–I15, N26.2
Atrial fibrillation	427.3x	I48
Peripheral arterial disease	440.x, 441.x, 443.x, 444.0x, 444.8x, 447.8x, 447.9x, 093.0, 437.3, 444.22, 447.1, 557.1, 557.9, V434	I70, I71, I73, I75, I771, I790, I791, I792, I773, I779, I798, K551, K558, K559, Z958, Z959, I743, I744, I745, I748, I740, I7789
Myocardial infarction	410.x, 412.x	I21-I22
Hemorrhage stroke	430.x–432.x	I60-I62
Ischemic stroke	433.xx–437.xx	I66, I651, I650, I658, I659, I636, I638, I639, G450, G458, G451, G452, G460, G461, G462, G459, G454, G463, G464, G465, G466, G467, G468, I670, I671, I672, I674, I675, I676, I677, I679, I680, I682, I688, I6302, I6312, I6322, I6521, I6522, I6523, I6529, I6359, I6309, I6319, I6300, I6310, I6320, I6329, I6330, I6339, I6340, I6349, I6350, I6359, I6789, I6781, I6782, I6789, I63031, I63032, I63039, I63131, I63132, I63139, I63231, I63232, I63239, I63011, I63012, I63019, I63111, I63112, I63119, I63211, I63212, I63219, I63311, I63312, I63319, I63321, I63322, I63329, I63331, I63332, I63339, I63341, I63342, I63349, I63411, I63412, I63419, I63421,

Disease	ICD-9-CM disease code	ICD-10-CM disease code
		I63422, I63429, I63431, I63432, I63439, I63441, I63442, I63449, I63511, I63512, I63519, I63521, I63522, I63529, I63531, I63532, I63539, I63541, I63542, I63549, I67841, I67848, I6331, I6333, I6334, I6341, I6342
Gout	274.x	M10, M1A.0, M1A.2, M1A.3, M1A.4, M1A.9, N20.0
Liver cirrhosis	571.2x, 571.5x, 571.6x	K70.30, K74.0, K74.60, K74.69, K74.3, K74.4, K74.5
Hepatitis B virus infection	070.20, 070.22, 070.30, 070.32, V02.61	B18.0, B18.1, B16.2, B19.11, B16.9, B19.10
Hepatitis C virus infection	070.41, 070.44, 070.51, 070.54, 070.70, 070.71, V02.62	B18.2, B17.10, B17.11, B19.20, B19.21

Abbreviation: ICD-9-CM, International Classification of Diseases, Ninth Revision, Clinical Modification; ICD-10-CM, International Classification of Diseases, Tenth Revision, Clinical Modification.

Supplementary Table S3. Complete baseline demographics and clinical characteristics at PD initiation of patients with and without exposure to icodextrin

Variable	Available number	Total (*n* = 1 800)	Icodextrin users (*n* = 1 102)	Icodextrin non-users (*n* = 698)	*P* value
Male	1 800	878 (48.8)	578 (52.5)	300 (43.0)	<0.001
Age, year	1 800	55.8 ± 15.1	55.4 ± 14.6	56.6 ± 15.9	0.104
Body mass index, kg/m^2	1 669	24.3 ± 4.5	24.9 ± 4.6	23.2 ± 4.2	<0.001
Comorbidity					
Diabetes mellitus	1 800	859 (47.7)	637 (57.8)	222 (31.8)	<0.001
Hypertension	1 800	1 627 (90.4)	1 029 (93.4)	598 (85.7)	<0.001
Atrial fibrillation	1 800	93 (5.2)	64 (5.8)	29 (4.2)	0.123
Peripheral artery disease	1 800	126 (7.0)	74 (6.7)	52 (7.4)	0.552
Myocardial infarction	1 800	113 (6.3)	77 (7.0)	36 (5.2)	0.119
Stroke	1 800	146 (8.1)	93 (8.4)	53 (7.6)	0.522
Gout	1 800	221 (12.3)	132 (12.0)	89 (12.8)	0.627
Liver cirrhosis	1 800	75 (4.2)	43 (3.9)	32 (4.6)	0.480
Hepatitis B virus infection	1 800	114 (6.3)	64 (5.8)	50 (7.2)	0.250
Hepatitis C virus infection	1 800	68 (3.8)	49 (4.4)	19 (2.7)	0.062
Cardiovascular disease	1 800	604 (33.6)	386 (35.0)	218 (31.2)	0.097
Left ventricular ejection fraction ≤60%	1 800	584 (32.4)	380 (34.5)	204 (29.2)	0.020
Primary renal disease	1 800				<0.001
Chronic tubulointerstitial disease		52 (2.9)	24 (2.2)	28 (4.0)	

Variable	Available number	Total (*n* = 1 800)	Icodextrin users (*n* = 1 102)	Icodextrin non-users (*n* = 698)	*P* value
Obstructive nephropathy		30 (1.7)	16 (1.5)	14 (2.0)	
Adult polycystic kidney disease		25 (1.4)	9 (0.8)	16 (2.3)	
Hypertension		912 (50.7)	523 (47.5)	389 (55.7)	
Diabetes mellitus		626 (34.8)	476 (43.2)	150 (21.5)	
Chronic glomerulonephritis		80 (4.4)	33 (3.0)	47 (6.7)	
Others		75 (4.2)	21 (1.9)	54 (7.7)	
Dialysis modality	1 800				0.037
APD		777 (43.2)	497 (45.1)	280 (40.1)	
CAPD		1 023 (56.8)	605 (54.9)	418 (59.9)	
Admissions in the previous year	1 800				0.279
0		1 588 (88.2)	965 (87.6)	623 (89.3)	
1-2		212 (11.8)	137 (12.4)	75 (10.7)	
Laboratory data at baseline					
Albumin, g/dL	1 787	3.49 ± 0.53	3.46 ± 0.50	3.54 ± 0.57	0.002
Hemoglobin, g/dL	1 796	9.57 ± 1.42	9.49 ± 1.34	9.70 ± 1.52	0.002
HbA1c, %	1 386	6.1 ± 1.2	6.2 ± 1.2	5.8 ± 1.0	<0.001
Low-density lipoprotein, mg/dL	1 297	108.4 ± 44.9	108.4 ± 45.3	108.4 ± 44.1	0.984
High-density lipoprotein, mg/dL	1 249	47.8 ± 16.4	47.5 ± 16.6	48.3 ± 16.3	0.453
Total cholesterol, mg/dL	1 741	184.2 ± 50.3	182.5 ± 49.7	187.0 ± 51.2	0.073
Triglyceride, mg/dL	1 740	149.5 ± 107.4	146.5 ± 96.4	154.5 ± 123.6	0.132
Uric acid, mg/dL	1 648	7.0 ± 2.0	6.9 ± 2.0	7.2 ± 2.0	0.006

Variable	Available number	Total (n = 1 800)	Icodextrin users (n = 1 102)	Icodextrin non-users (n = 698)	P value
Medication at baseline					
High potency statin	1 800	81 (4.5)	58 (5.3)	23 (3.3)	0.050
ACEi/ARB	1 800	1 317 (73.2)	849 (77.0)	468 (67.0)	<0.001
Beta-blocker	1 800	732 (40.7)	486 (44.1)	246 (35.2)	<0.001
Calcium-channel blocker	1 800	1 293 (71.8)	840 (76.2)	453 (64.9)	<0.001
Loop diuretics	1 800	1 259 (69.9)	824 (74.8)	435 (62.3)	<0.001
Mineralocorticoid receptor antagonists	1 800	110 (6.1)	78 (7.1)	32 (4.6)	0.031
Nitrate	1 800	537 (29.8)	340 (30.9)	197 (28.2)	0.235
Other vasodilators	1 800	331 (18.4)	227 (20.6)	104 (14.9)	0.002
ARNI	1 800	10 (0.6)	8 (0.7)	2 (0.3)	0.222
Antiplatelet agent	1 800	590 (32.8)	395 (35.8)	195 (27.9)	<0.001
Insulin	1 800	523 (29.1)	391 (35.5)	132 (18.9)	<0.001
Oral antidiabetic drug	1 800	585 (32.5)	446 (40.5)	139 (19.9)	<0.001
Cigarette smoking	1 800	309 (17.2)	213 (19.3)	96 (13.8)	0.002
Alcohol drinking	1 800	158 (8.8)	105 (9.5)	53 (7.6)	0.157
Year of dialysis initiation	1 800				<0.001
2005-2010		535 (29.7)	283 (25.7)	252 (36.1)	
2011-2016		643 (35.7)	412 (37.4)	231 (33.1)	
2017-2022		622 (34.6)	407 (36.9)	215 (30.8)	
Duration from onset of CKD to PD, year	1 476	4.2 ± 3.9	4.0 ± 3.8	4.5 ± 4.0	0.024
Follow up years	1 800	3.5 ± 3.1	3.6 ± 2.9	3.2 ± 3.2	0.006

Abbreviation: APD, automated peritoneal dialysis; CAPD, continuous ambulatory peritoneal dialysis; HbA1c, glycated hemoglobin; ACEi, angiotensin converting enzyme inhibitor; ARB, angiotensin receptor blocker; ARNI, angiotensin receptor-neprilysin inhibitor;
Data are presented as frequency (percentage), mean ± standard deviation or median [25th, 75th percentiles].

Supplementary Table S4. The incidence of outcomes

Outcome	No. of event (%)	Incidence† (95% CI)
All-cause death	480 (26.7)	7.7 (7.0–8.4)
Cardiovascular death	126 (7.0)	2.0 (1.7–2.4)
Sudden death	370 (20.6)	5.9 (5.3–6.5)
Heart failure hospitalization	358 (19.9)	4.7 (4.2–5.2)
Myocardial infarction	185 (10.3)	2.1 (1.8–2.4)
Ischemic stroke	163 (9.1)	1.8 (1.6–2.1)
Major adverse cardiovascular events (MACE)#	523 (29.1)	9.1 (8.4–9.9)
New diagnosis of cancer	252 (14.0)	3.2 (2.8–3.5)
Encapsulated peritoneal sclerosis	301 (16.7)	4.0 (3.5–4.4)
Shift to hemodialysis	779 (43.3)	19.5 (18.2–20.9)
Renal transplantation	98 (5.4)	1.1 (0.9–1.3)

Abbreviation: CI, confidence interval;

† Number of events per 100 person-year.

# Any of cardiovascular death, heart failure hospitalization, myocardial infarction and ischemic stroke.

Supplementary Table S5. Outcomes compared between icodextrin users and non-users with an alternative time-dependent exposure

	Incidence† (95% CI)		Crude analysis		Multivariable analysis‡	
Outcome	Exposed (n = 13 075)	Unexposed (n = 23 545)	HR (95% CI)	*P* value	HR (95% CI)	*P* value
All-cause death	1.1 (0.7–1.5)	7.1 (6.4–7.9)	0.15 (0.11–0.22)	<0.001	0.14 (0.10–0.19)	<0.001
Cardiovascular death	0.2 (0.0–0.3)	2.0 (1.6–2.4)	0.09 (0.04–0.21)	<0.001	0.08 (0.04–0.18)	<0.001
Sudden death	0.9 (0.6–1.3)	5.4 (4.8–6.0)	0.18 (0.12–0.26)	<0.001	0.16 (0.11–0.23)	<0.001
Heart failure hospitalization	3.8 (3.1–4.5)	2.6 (2.2–3.1)	1.49 (1.15–1.93)	0.003	1.15 (0.88–1.50)	0.323
Myocardial infarction	1.7 (1.2–2.1)	1.2 (0.9–1.5)	1.40 (0.97–2.04)	0.076	1.12 (0.76–1.64)	0.573
Ischemic stroke	1.4 (1.0–1.8)	1.0 (0.8–1.3)	1.37 (0.92–2.05)	0.122	1.40 (0.93–2.12)	0.109
MACE#	6.0 (5.1–6.9)	6.9 (6.2–7.7)	0.86 (0.71–1.04)	0.119	0.69 (0.57–0.84)	<0.001
New diagnosis of cancer	0.9 (0.6–1.2)	2.2 (1.8–2.6)	0.41 (0.27–0.61)	<0.001	0.38 (0.25–0.58)	<0.001
Encapsulated peritoneal sclerosis	2.3 (1.8–2.9)	3.8 (3.3–4.4)	0.60 (0.46–0.79)	<0.001	0.62 (0.47–0.81)	<0.001
Shift to hemodialysis	4.8 (4.1–5.6)	18.6 (17.0–20.1)	0.26 (0.22–0.31)	<0.001	0.21 (0.17–0.25)	<0.001
Renal transplantation	0.2 (0.0–0.3)	1.7 (1.4–2.1)	0.11 (0.05–0.24)	<0.001	0.10 (0.04–0.23)	<0.001

Abbreviation: CI, confidence interval; HR, hazard ratio; MACE, major adverse cardiac event;

† Number of events per 100 person-year;

‡ Adjusted for sex, age, body mass index, diabetes mellitus, hypertension, cardiovascular disease, left ventricular ejection fraction, dialysis modality, admissions in the previous year, albumin, hemoglobin, high potency statin, loop diuretics, insulin, cigarette smoking, alcohol drinking and year of dialysis initiation;

# Any of cardiovascular death, heart failure hospitalization, myocardial infarction and ischemic stroke.

Supplementary References

1. Mistry CD, Gokal R, Peers E. A randomized multicenter clinical trial comparing isosmolar icodextrin with hyperosmolar glucose solutions in CAPD. MIDAS Study Group. Multicenter Investigation of Icodextrin in Ambulatory Peritoneal Dialysis. *Kidney Int* 1994;**46**(2):496-503
2. Gokal R, Mistry CD, Peers EM. Peritonitis occurrence in a multicenter study of icodextrin and glucose in CAPD. MIDAS Study Group. Multicenter Investigation of Icodextrin in Ambulatory Dialysis. *Perit Dial Int* 1995;**15**(6):226-30
3. Posthuma N, ter Wee PM, Verbrugh HA *et al.* Icodextrin instead of glucose during the daytime dwell in CCPD increases ultrafiltration and 24-h dialysate creatinine clearance. *Nephrol Dial Transplant* 1997;**12**(3):550-3
4. Posthuma N, ter Wee PM, Donker AJ *et al.* Serum disaccharides and osmolality in CCPD patients using icodextrin or glucose as daytime dwell. *Perit Dial Int* 1997;**17**(6):602-7
5. Posthuma N, ter Wee PM, Donker AJ *et al.* Assessment of the effectiveness, safety, and biocompatibility of icodextrin in automated peritoneal dialysis. The Dextrin in APD in Amsterdam (DIANA) Group. *Perit Dial Int* 2000;**20 Suppl 2**:S106-13
6. Bredie SJ, Bosch FH, Demacker PN *et al.* Effects of peritoneal dialysis with an overnight icodextrin dwell on parameters of glucose and lipid metabolism. *Perit Dial Int* 2001;**21**(3):275-81
7. Plum J, Gentile S, Verger C *et al.* Efficacy and safety of a 7.5% icodextrin peritoneal dialysis solution in patients treated with automated peritoneal dialysis. *Am J Kidney Dis* 2002;**39**(4):862-71
8. Wolfson M, Piraino B, Hamburger RJ *et al.* A randomized controlled trial to evaluate the efficacy and safety of icodextrin in peritoneal dialysis. *Am J Kidney Dis* 2002;**40**(5):1055-65
9. Davies SJ, Woodrow G, Donovan K *et al.* Icodextrin improves the fluid status of peritoneal dialysis patients: results of a double-blind randomized controlled trial. *J Am Soc Nephrol* 2003;**14**(9):2338-44
10. Konings CJ, Kooman JP, Schonck M *et al.* Effect of icodextrin on volume status, blood pressure and echocardiographic parameters: a randomized study. *Kidney Int* 2003;**63**(4):1556-63
11. Finkelstein F, Healy H, Abu-Alfa A *et al.* Superiority of icodextrin compared with 4.25% dextrose for peritoneal ultrafiltration. *J Am Soc Nephrol* 2005;**16**(2):546-54

12. Rodríguez-Carmona A, Pérez Fontán M, García López E *et al.* Use of icodextrin during nocturnal automated peritoneal dialysis allows sustained ultrafiltration while reducing the peritoneal glucose load: a randomized crossover study. *Perit Dial Int* 2007;**27**(3):260-6
13. Davies SJ, Garcia Lopez E, Woodrow G *et al.* Longitudinal relationships between fluid status, inflammation, urine volume and plasma metabolites of icodextrin in patients randomized to glucose or icodextrin for the long exchange. *Nephrol Dial Transplant* 2008;**23**(9):2982-8
14. Paniagua R, Orihuela O, Ventura MD *et al.* Echocardiographic, electrocardiographic and blood pressure changes induced by icodextrin solution in diabetic patients on peritoneal dialysis. *Kidney Int Suppl* 2008(108):S125-30
15. Lin A, Qian J, Li X *et al.* Randomized controlled trial of icodextrin versus glucose containing peritoneal dialysis fluid. *Clin J Am Soc Nephrol* 2009;**4**(11):1799-804
16. Paniagua R, Ventura MD, Avila-Díaz M *et al.* Icodextrin improves metabolic and fluid management in high and high-average transport diabetic patients. *Perit Dial Int* 2009;**29**(4):422-32
17. Qi H, Xu C, Yan H *et al.* Comparison of icodextrin and glucose solutions for long dwell exchange in peritoneal dialysis: a meta-analysis of randomized controlled trials. *Perit Dial Int* 2011;**31**(2):179-88
18. Takatori Y, Akagi S, Sugiyama H *et al.* Icodextrin increases technique survival rate in peritoneal dialysis patients with diabetic nephropathy by improving body fluid management: a randomized controlled trial. *Clin J Am Soc Nephrol* 2011;**6**(6):1337-44
19. Han SH, Ahn SV, Yun JY *et al.* Effects of icodextrin on patient survival and technique success in patients undergoing peritoneal dialysis. *Nephrol Dial Transplant* 2012;**27**(5):2044-50
20. Chow KM, Szeto CC, Kwan BC *et al.* Randomized controlled study of icodextrin on the treatment of peritoneal dialysis patients during acute peritonitis. *Nephrol Dial Transplant* 2014;**29**(7):1438-43
21. Sniderman AD, Sloand JA, Li PK *et al.* Influence of low-glucose peritoneal dialysis on serum lipids and apolipoproteins in the IMPENDIA/EDEN trials. *J Clin Lipidol* 2014;**8**(4):441-7
22. Yoon HE, Chang YK, Shin SJ *et al.* Benefits of a continuous ambulatory peritoneal dialysis (CAPD) technique with one icodextrin-containing and two biocompatible glucose-containing dialysates for preservation of residual renal function and biocompatibility in incident CAPD patients. *J Korean Med Sci* 2014;**29**(9):1217-25
23. de Moraes TP, Andreoli MC, Canziani ME *et al.* Icodextrin reduces insulin resistance in non-diabetic patients undergoing automated peritoneal dialysis: results of a randomized controlled trial (STARCH). *Nephrol Dial Transplant* 2015;**30**(11):1905-11

24. Chang TI, Ryu DR, Yoo TH *et al.* Effect of Icodextrin Solution on the Preservation of Residual Renal Function in Peritoneal Dialysis Patients: A Randomized Controlled Study. *Medicine (Baltimore)* 2016;**95**(13):e2991
25. Chen JB, Cheng BC, Liu WH *et al.* Longitudinal analysis of cardiac structure and function in incident-automated peritoneal dialysis: comparison between icodextrin solution and glucose-based solution. *BMC Nephrol* 2018;**19**(1):109
26. Htay H, Johnson DW, Wiggins KJ *et al.* Biocompatible dialysis fluids for peritoneal dialysis. *Cochrane Database Syst Rev* 2018;**10**(10):CD007554
27. Wang IK, Lin CL, Yen TH *et al.* Icodextrin reduces the risk of congestive heart failure in peritoneal dialysis patients. *Pharmacoepidemiol Drug Saf* 2018;**27**(4):447-52
28. Goossen K, Becker M, Marshall MR *et al.* Icodextrin Versus Glucose Solutions for the Once-Daily Long Dwell in Peritoneal Dialysis: An Enriched Systematic Review and Meta-analysis of Randomized Controlled Trials. *Am J Kidney Dis* 2020;**75**(6):830-46
29. Cordeiro L, Ishikawa WY, Andreoli MCC *et al.* A randomized clinical trial to evaluate the effects of icodextrin on left ventricular mass index in peritoneal dialysis. *Sci Rep* 2022;**12**(1):15776
30. Azevedo LS, Banin VB, Dias DB *et al.* Icodextrin versus Glucose 2.5% on markers of hypervolemia and survival of patients undergoing automated peritoneal dialysis with an unplanned start: a randomized controlled trial. *Einstein (Sao Paulo)* 2024;**22**:eAO0980
31. Wang IK, Chan CI, Lin AH *et al.* The impact of icodextrin on the outcomes of incident peritoneal dialysis patients. *PLoS One* 2024;**19**(3):e0297688
